# Psychometric properties of online administered parental strengths and difficulties questionnaire (SDQ), and normative data based on combined online and paper-and-pencil administration

**DOI:** 10.1186/1753-2000-7-40

**Published:** 2013-12-10

**Authors:** Annika Björnsdotter, Pia Enebrink, Ata Ghaderi

**Affiliations:** 1 Department of Psychology, Uppsala University, Box 1225, SE-75142 Uppsala, Sweden; 2 Department of Clinical Neuroscience, Division of Psychology, Karolinska Institutet, SE-17177 Stockholm, Sweden

**Keywords:** Psychometrics, Conduct problems, Online assessment, Norms, Disruptive behavior disorder

## Abstract

**Objective:**

To examine the psychometric properties of the online administered parental version of the Strength and Difficulties Questionnaire (SDQ), and to provide parental norms from a nationwide Swedish sample.

**Methods:**

A total of 1443 parents from of a national probability sample of 2800 children aged 10-13 years completed the SDQ online or as usual (i.e., using paper-and-pencil).

**Results:**

The SDQ subscales obtained from the online administration showed high internal consistency (polychoric ordinal alpha), and confirmatory factor analysis of the SDQ five factor model resulted in excellent fit. The Total Difficulties score of the SDQ and its other subscales were significantly related to the Disruptive Behavior Disorders (DBD) rating scale. Norms for the parent version of SDQ obtained from the Internet were identical to those collected using paper-and-pencil. They were thus combined and are presented sorted by child gender and age.

**Conclusions:**

The SDQ seems to be a reliable and valid instrument given its high internal consistency, clear factor structure and high correlation with other instruments capturing the intended constructs. Findings in the present study support its use for online data collection, as well as using norms obtained through paper-and-pencil-administration even when SDQ has been administrated online.

## Introduction

Annual or cumulative prevalence rate of mental health problems and psychiatric illness among youth are high (e.g., [1-3]). Children with early onset of either internalizing problems such as anxiousness and withdrawn behavior (e.g., [4]) or persistent externalizing problems such as defiant and disruptive behavior (e.g., [5-7]) are also at higher risk for continued severe and debilitating mental health problems during adolescence and adulthood [8,9]. Early interventions in terms of parent management training (PMT) programs (e.g., [10-12]) for troubled children have been found to reduce the risk for developing later mental health problems. To reliably identify children with elevated risk for continued externalizing and internalizing problems, and to be able to evaluate the effectiveness of interventions targeting these children and their parents, we need reliable and valid instruments.

Most available instruments with strong psychometrics in this context focus heavily on children’s difficulties only (e.g., Child Behavior Checklist, CBCL: [13]), instead of capturing both strengths and problems. The Strengths and Difficulties Questionnaire SDQ: [14-16] is a well-known, frequently used instrument in research [17,18], that can be completed in five minutes by parents or teachers for assessing psychological problems and prosocial behaviors among children aged 3–16 years. A unique aspect of the SDQ is its focus on not only problems, but also on the strengths. Scores derived from the SDQ are highly correlated with CBCL, and it is significantly better than the CBCL in detecting inattention and hyperactivity when they both are compared to a semi-structured interview [19]. The SDQ has been evaluated widely internationally and is considered to be an instrument with good psychometrics (for a review, see [20]). Although some studies have replicated its original factor structure (e.g., [21]), other studies have failed to reproduce the same factor structure (e.g., [22-24]), and normative data for parent-ratings of older children in Sweden are missing.

Increased use of online data collection in both screening and intervention studies, as well as in clinical practice, raises new questions about the psychometrics of instruments administered online and applicability of norms obtained through traditional mode of data collection (i.e., paper-and-pencil). Some systematic differences have been noted in response to the questionnaire administered online versus on paper-and-pencil [25,26]. Joinson [27] found that people consistently gave higher severity rating on their online report compared to paper-and-pencil. However, most studies have found high correlations between scores obtained from these different modes of data collection and that the clinical relevance of found discrepancies are generally negligible (e.g., [28-31]). Research on the psychometrics of instruments measuring child behaviors online is scarce, and that is especially true for the SDQ. Furthermore, we need to know whether norms obtained from the online surveys are different from those collected by paper-and-pencil. Last, but not least, Swedish norms for children and adolescents aged 10–13 are not yet available, and thus needed. Consequently, the aim of the present study was to 1) examine the psychometric properties of the online administered parental rating of the SDQ in terms of its internal consistency, factor structure, and concurrent validity with another instrument measuring similar constructs, and 2) provide parental norms for the SDQ from a nationwide representative Swedish sample of 10–13 year old children.

## Methods

### Participants

By stratifying based on children’s gender at each age (10–13 years), the Swedish Population Address Register (SPAR) provided a random sample selected across the entire Sweden, with adequate distribution and representation of both sexes at each age interval. A total of 2800 children were randomly selected and their parents were asked to complete a survey including the SDQ and several other self-report questionnaires. Ten parents could not be reached due to unknown addresses, and eight were excluded due to language difficulties. No demographics were obtained from parents who actively declined to participate or parents who ignored to respond to reminders about participation. The only available information on all the parents was their complete addresses. No significant differences with an effect size larger than Cohen’s *d* = 0.10 emerged when respondents were compared with those who actively declined participation and those who ignored the study, and the combination of the latter two groups (non-participants) based on variables derived from their postal addresses. The available socio demographic variables (i.e., rural verses urban, south versus mid or north part of Sweden) showed no significant differences. The parent’s educational level was considerably higher than the average educational level of parents in Sweden. Thirty-two percent of the total population of Sweden has a college/university degree, but 53.7% of the study sample reported having such a degree. On the other hand, 44% of the total population has a high school degree, which is in line with the corresponding figure in our study (39.9%). Compared to the total population (with an age span of 16 – 75 years) where only 34% are married, the responding parents were also to a larger extent married (64%). This very large difference is most probably a consequence of the fact that the study sample is based on children between the ages of 10 and 13. This might most probably lead to selection of parents that are married or cohabitant to a larger degree than what would be the case in the general population of all adults in Sweden.

A total of 1443 parents completed the questionnaires (457 responded online and 986 via paper-and-pencil). Ten parents were excluded from the analyses as they failed to report their gender/role (mother or father), yielding a total response rate of 51.2% (31.9% online and 68.1% paper-and-pencil). Of all the respondents, 52.8% were mothers and 47.2% fathers. In addition, two stepparents responded to the questionnaire. They were coded as mothers and fathers, respectively in the analysis. The marital status of parents as well as their educational level, and children’s gender are shown in Table 1.

**Table 1:** Characteristics of the sample

	**Internet *n*** **= 457**	**Paper-and-pencil *n*** **= 976**	**Total sample *n*** **= 1433**
**Parents’ marital status**	Number (%)	Number (%)	Number (%)
Married	307 (67.2)	604 (61.9)	911 (63.6)
Single-parents	54 (11.8)	125 (12.8)	179 (12.5)
Living together, but not married	85 (18.6)	207 (21.2)	292 (20.4)
Other	11 (2.4)	33 (3.4)	44 (3.1)
Missing value	-	7 (0.7)	7 (0.5)
**Education**			
Elementary school or less	20 (4.4)	71 (7.3)	91 (6.4)
High school	190 (41.6)	380 (38.9)	570 (39.8)
College or university	247 (54.0)	521 (53.4)	768 (53.6)
Missing values	-	4 (0.4)	4 (0.3)
**Children’s gender**			
Girls	240 (52.5)	483 (49.5)	723 (50.5)
Boys	217 (47.5)	493 (50.5)	710 (49.5)

### Instruments

The SDQ is, as mentioned, a brief screening instrument for behavioral and emotional problems in children and adolescents. The SDQ items were initially selected on the basis of relevant concepts as well as factor analysis [32]. A parent and a teacher form of the SDQ are available for children aged 3–16 years, and a youth report form is available for the age span 11–16 years. The SDQ symptom scales contain 25 items divided into five subscales, namely Emotional Symptoms, Conduct Problems, Hyperactivity-Inattention, Peer Problems, and Prosocial Behavior. A 3-point Likert-type scale is employed to indicate how each attribute applies to the target child (0 = *not true*, 1 = *somewhat true*, 2 = *certainly true*). Some of the items are reversed. A high score on the Prosocial Behavior subscale reflects strength, while high scores on the other four SDQ subscales reflect difficulties. All subscales but Prosocial Behaviors are also summed together to generate the Total Difficulties score. The SDQ also includes an impact scale to score to what extent the child has a problem with emotions, concentration, or with how to get on with other people. The SDQ also contains four questions about chronicity, distress, social impairment, and possible burden to others. The scoring algorithms allow the subscale scores to be prorated if at least three of the five subscale items are complete (http://www.sdqinfo.org). Factor analytic studies have shown mixed results across countries. The five psychological dimensions of the SDQ have been confirmed in studies, among others in Sweden [21], UK [32], and Germany [33]. Exploratory factor analysis of the US NHIS data, has however found that the best-fitting factor solution involved only three dimensions. Those were externalizing, internalizing, and a prosocial dimension [22].

The Disruptive Behavior Disorders (DBD) rating scale [7] can be responded to by parents or teachers. The DBD covers the DSM-IV-based symptoms [34] for all three disruptive behavior disorders: Attention Deficit/Hyperactivity Disorder (ADHD: 18 items), Oppositional Defiant Disorder (ODD: 8 items) and Conduct Disorder (CD: 15 items). Each item is rated on a 4-point Likert-type scale (0 = *not at all*, 1 = *just a little*, 2 *= pretty much*, and 3 = *very much*). The DBD rating scale includes 45 items. After the revision of the DSM-III-R to DSM-IV [34,35], three items are no longer coded in the scoring (item 10, 14 and 21). Item 5 (*Often initiates physical fights with other members of his or her household*) does not correspond to any criteria in either the DSM-III-R or the DSM-IV, and is not coded. The responses on the DBD can be summarized using “symptom count” or “composite scores”. For the present study, composite scores were calculated by adding the items within each subscale [7]. The internal consistency (polychoric ordinal alpha: Please see Statistical analysis) of the subscales of the DBD varied between .97 and .99. When the internal consistency was calculated for boys versus girls, mothers versus fathers or the Internet versus paper-and-pencil, very small differences emerged, and the range was still within the upper limits (.94 to .99).

### Procedure

An invitation letter was sent to all the 2800 families detailing the purpose and procedures of the study. They were informed that they would be randomly assigned to respond to the questionnaire using the Internet or via paper-and-pencil. They were also asked to return a form using an enclosed pre paid envelope in case they chose not to participate in the study, or if they would prefer to participate under the condition of using paper-and-pencil. A total of 462 responses of refusal were received, and 170 parents indicated that they preferred to respond to the questionnaire on paper. Out of these 170 parents, 142 had already been randomized to paper-and-pencil condition and 28 the Internet. A reminder letter was sent out to the parents within 4 weeks. To increase the response rate, parents who had not responded were also reminded through a phone call. Within eight to twelve weeks from the first letter, a second reminder letter along with the questionnaires was sent out.

Of all the responses, 31.9% was from the online administration, and 68.1% from the paper-and-pencil condition.

Parents who responded to the questionnaire had the opportunity to choose among three different small gifts. The options were two cinema tickets or an equal amount of money (approximately 30 USD) in terms of shopping gift certificates to be used via the Internet, or by donating the gratification to a Child Cancer Foundation. The project was approved by the Regional Ethical Board (dnr 2010119).

### Statistical analysis

The Statistical Package for the Social Sciences (SPSS version 19) was used for the main analysis.

There were less than one percent non-systematic missing values for the single 25 first items of the SDQ and less than 1.2% missing values on the DBD single items. Since the subscale scores of the SDQ can be prorated if at least 3 items are completed, the missing values were not replaced. Similarly, the missing values on the DBD were not replaced either, although the rate of missing data was at a slightly higher rate. The rationale for this procedure was the fact that in response to the DBD parents and teachers are allowed to indicate that they don’t know the answer to some questions due to lack of information.

Chi-square, t-tests and ANOVAs were used to explore possible differences in categorical and continuous background variables. To explore whether parents with different levels of education and marital status (both with more than two conditions) responded significantly different to the SDQ, multiple group comparisons after significant F-test were done using Bonferroni correction. Cohen’s *d* or partial eta squared was used as a measure of effect size for group comparisons. Due to considerable to very high skewness and/or kurtosis on a number of items in the SDQ, and given the response format (3 points only), polychoric ordinal alpha [36] was calculated instead of Cronbach’s alpha. Polychoric correlations between the items in each subscale were first obtained from PRELIS [37]. The average correlation (r_average_) was then entered into the formula provided by Gadermann et al. (2012), where k is the number of items in the scale:

$$\text{Polychoric}\;\text{ordinal}\;\text{alpha}=\left({k*r}_{\text{average}}\right)/\left(1+\left(k‒1\right)*r_{\text{average}}\right)$$

Through confirmatory factor analysis (CFA) the fit of the Goodman’s theoretical model of the SDQ comprising five factors [14] was investigated. For the CFA, LISREL 9 [38] was used. The global model fit to the data was tested by Chi-square, Root Mean Square Error of Approximation (RMSEA), Comparative Fit Index (CFI) and Goodness of Fit Index (GFI). The alpha was set to *p* < .05.

## Results

### Psychometrics of SDQ online data

#### Missing values and characteristics at item level

Scrutinizing the SDQ at item level showed low rate of missing values (0.03% to 1%). Item 12 (*Often fights with other children or bullies them*), item 17 (*Kind to younger children*), and item 22 (*Steals from home, school or elsewhere*) had all high skewness (4.9, -3.7, and 6.3, respectively). In addition, item 12, 17, and 22 also showed very high kurtosis (i.e., 26.0, 14.1, and 42.7, respectively).

#### The internal consistency

The internal consistency (polychoric ordinal alpha) based on the online data (*N* = 457) of the SDQ was high, ranging from .85 to .91 (Emotional Problems: .89, Hyperactivity-Inattention: .89, Peer Problems: .85, Prosocial Behavior: .91, and Conduct Problems: .89). The internal consistency based on data from mothers (*n* = 243), or fathers (*n* = 214), as well data regarding daughters (*n* = 240) versus sons (*n* = 217) were virtually identical (ranging from .84 to .91).

#### The factor structure

**Figure 1:**
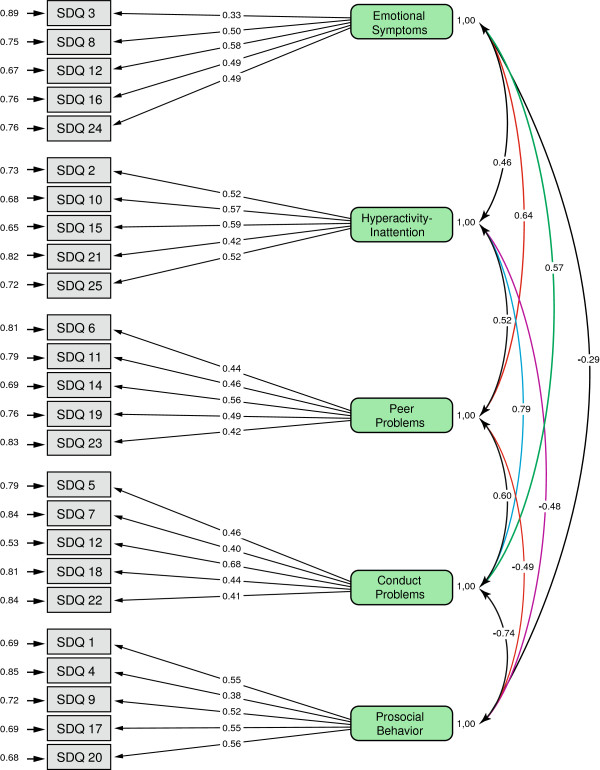
Confirmatory factor analysis of the SDQ from online data.

CFA of the SDQ (Figure 1) for data from the online administration resulted in excellent fit (*χ*^2^ = 413.45, *p* < .001, RMSEA = .035, 90% CI for RMSEA = (0.213 - 0.455), GFI = .93, and CFI = .96). The model showed similar excellent fit for mothers, (*χ*^2^ = 360.3, *p* < .001, RMSEA = .012, 90% CI for RMSEA = (0.0 - 0.029), GFI = .92, and CFI = .95), but the fit indices showed considerable lack of fit for fathers. Scrutinizing data showed that item 22 *(Steals from home, school or elsewhere*) was the source of problem. Rerunning the CFA without item 22 resulted in excellent fit (*χ*^2^ = 392.23, *p* < .001, RMSEA = .017, 90% CI for RMSEA = (0.0 - 0.034), GFI = .91, and CFI = .91). Rerunning the analysis for boys only lead to excellent fit (RMSEA = 0.0, and 90% CI for RMSEA = (0.0 - 0.0), GFI = .99, and CFI = .99. The CFA for girls only resulted in similar fit indices (RMSEA = .025, and 90% CI for RMSEA = (0.005 - 0.036), GFI = .91, and CFI = .94).

#### Concurrrent validity of the SDQ

The subscales Hyperactivity–Inattention and Conduct Problems as well as the score of Total Difficulties of the SDQ and the subscales of DBD, were related to each other significantly and meaningfully, as can be seen in Table 2. The correlations for the online sample (*N* = 454–456) were all significant at *p* < .001, which means that they would remain significant even after Bonferroni correction.

**Table 2:** The Pearson correlations between the SDQ and the DBD from data administered online

	**DBD**			
	**Inattention**	**Hyperactivity/Impulsivity**	**ODD**^**1**^	**CD**^**2**^
SDQ				
Emotional Symptoms	.33	.32	.33	.12
Hyperactivity-Inattention	.64	.62	.45	.28
Peer Problems	.36	.33	.34	.17
Conduct Problems	.47	.58	.66	.40
Prosocial Behavior	-.36	-.36	-.38	-.24
Total Difficulties	.63	.63	.59	.32

^1^ODD = Oppositional Defiant Disorders subscale of the DBD.

^2^CD = Conduct Disorder subscale of the DBD.

Prosocial Behavior was negatively correlated to all the subscales of the DBD, while other subscales of the SDQ correlated positively, as expected, with the DBD. The subscale Emotional Problems and Peer Problems were correlated, however to a lesser extent, to all DBD subscales which was in line with expectations.

### Swedish norms for children in the age span of 10 to 13 years

#### Parental response through the Internet versus on paper

The mean scores of the SDQ Total Difficulties and the other subscales from mothers or fathers, as well as all parents together responding online were not significantly different from mean scores of equivalent group of parents responding through paper-and-pencil either for the entire group of children or for different child age and gender with one exception (Table 3).

**Table 3:** Mean (M), standard deviation (SD) and effect size of the difference in parental response (online versus on paper-and-pencil) to the SDQ

	**Online M (SD)**	**Paper-and-Pencil M (SD)**	**Effect size of the difference (*η***_**p**_^**2**^**)**
	**N = 437**	**N = 946**	
Emotional Symptoms	1.7 (1.8)	1.4 (1.7)	.003
Hyperactivity-Inattention	2.3 (2.1)	2.3 (2.1)	.000
Peer Problems	1.2 (1.5)	1.2 (1.5)	.000
Conduct Problems	1.0 (1.2)	1.1 (1.3)	.001
Prosocial Behavior	8.3 (1.7)	8.5 (1.6)	.001
Total Difficulties	6.2 (4.7)	6.1 (4.8)	.000

As shown in Table 3, the magnitude of effect concerning the difference in mean scores reported online versus on paper and pencil were virtually zero, as a partial eta-squared of .02 corresponds to a small effect and those in Table 3 were all below .004. The Hyperactivity-Inattention subscale of the SDQ reported by fathers of 10 years old girls (*M* = 2.84, *SD* = 1.95) via the Internet was significantly higher (*t*(75) = 2.51, *p* = .21, Cohen’s *d* = 0.60) than corresponding value from fathers using paper-and-pencil (*M* = 1.75, *SD* = 1.7). Given the low number of fathers (*n* = 25) of 10-years old girls responding via the Internet leading to increased risk for bias, presence of extreme values in the scores that violates the assumptions in the parametric analyses, and the fact that this finding would not remain after correction for multiple comparisons, all data were combined. That is, all responses (mothers and fathers) collected online as well as from paper-and-pencil were combined. No significant differences were found regarding the subscales of SDQ or the Total Difficulties score, between parents with different marital status, or level of education (recoded into high or low to increase the power in the analyses), with two exceptions. Parents with lower education reported significantly higher scores on the subscale Hyperactivity-Inattention (mean difference = 0.34) as well as higher SDQ Total Difficulties score (mean difference = 0.79). These differences correspond to an effect size of *d* = 0.16 and *d* = 0.15 respectively, which are considered as small effect sizes.

#### Norms including both modes of data collection

Since the norms obtained online were not significantly different from those based on paper-and-pencil, they were combined. Parental norms for the SDQ, based on children’s age and gender are reported in Table 4.

**Table 4:** General population-based parental norms for the SDQ

	**10-years old**		**11-years old**		**12-years old**		**13-years old**	
**Emotional Symptoms**	Girls *n* = 154	Boys *n* = 159	Girls *n* = 232	Boys *n* = 206	Girls *n* = 158	Boys *n* = 159	Girls *n* = 179	Boys *n* = 186
M (SD) Mdn*	1.6 (1.8) 1.0	1.3 (1.7) 1.0	1.5 (1.8) 1.0	1.6 (1.9) 1.0	1.7 (1.7) 1.0	1.6 (1.8) 1.0	1.7 (1.9) 1.0	1.4 (1.7)* 1.0
80th percentile	3.0	3.0	3.0	3.0	3.0	3.0	3.0	2.0
85th percentile	4.0	3.0	3.0	4.0	3.5	3.0	3.0	3.0
90th percentile	4.0	4.0	4.0	4.0	4.0	4.0	4.0	4.0
95th percentile	5.0	5.0	5.5	5.0	5.0	5.0	5.0	5.0
**Conduct Problems**								
M (SD) Mdn*	1.1 (1.4) 1.0	1.0 (1.2) 1.0	1.0 (1.3) 1.0	1.0 (1.2) 1.0	1.2 (1.2) 1.0	1.3 (1.6) 1.0	1.3 (1.4) 1.0	1.0 (1.2) 1.0
80th percentile	2.0	2.0	2.0	2.0	2.0	2.0	2.0	2.0
85th percentile	2.0	2.0	2.0	2.6	2.0	3.0	3.0	2.0
90th percentile	3.0	3.0	2.0	3.0	3.0	4.0	3.0	3.0
95th percentile	4.0	4.0	4.0	3.0	4.0	5.0	4.0	3.0
**Hyperactivity-Inattention**								
M (SD) Mdn*	2.1 (2.0) 2.0	2.6 (1.9) 2.0	2.1 (2.1) 2.0	2.7 (2.3) 2.0	2.1 (2.1) 2.0	2.6 (2.2) 2.0	2.1 (2.1) 2.0	2.5 (2.1) 2.0
80th percentile	3.2	4.0	4.0	5.0	4.0	4.0	4.0	4.0
85th percentile	4.0	5.0	4.0	5.0	4.5	5.0	4.0	5.0
90th percentile	5.0	5.0	5.0	6.0	5.0	6.0	5.0	5.0
95th percentile	6.0	6.0	6.5	7.0	7.0	7.0	6.2	7.0
**Peer Problems**								
M (SD) Mdn*	1.1 (1.8) 0.0	1.2 (1.7) 1.0	1.1 (1.5) 0.0	1.3 (1.5) 1.0	1.1 (1.5) 1.0	1.4 (1.9) 1.0	1.5 (1.8) 1.0	1.4 (1.7) 1.0
80th percentile	2.0	2.0	2.0	2.0	2.0	3.0	3.0	3.0
85th percentile	2.0	3.0	3.0	3.0	2.5	3.0	3.0	3.0
90th percentile	3.1	3.1	3.0	3.7	3.0	4.0	4.0	4.0
95th percentile	5.0	5.0	4.0	4.9	5.0	6.0	5.0	5.0
**Prosocial Behavior**								
M (SD) Mdn*	8.6 (1.5) 9.0	8.2 (1.9) 8.0	8.6 (1.6) 9.0	8.2 (1.7) 8.0	8.6 (1.5) 9.0	8.2 (1.9) 9.0	8.4 (1.7) 9.0	8.3 (1.7) 9.0
20th percentile	7.0	7.0	7.0	7.0	7.0	7.0	7.0	7.0
15th percentile	7.0	6.0	7.0	6.0	7.0	6.0	6.0	6.2
10th percentile	6.0	6.0	6.0	6.0	6.0	6.0	6.0	6.0
5th percentile	6.0	4.0	5.0	5.0	5.0	5.0	5.0	5.0
**Total Difficulties**								
M (SD) Mdn*	5.8 (5.2) 4.0	6.1 (4.6) 5.0	5.7 (5.0) 4.0	6.6 (5.1) 5.5	6.1 (4.7) 5.0	6.9 (6.0) 5.0	6.6 (5.5) 5.0	6.3 (4.8) 5.0
80th percentile	9.0	9.0	9.0	11.0	10.0	10.0	11.0	10.0
85th percentile	11.0	11.0	10.0	12.0	12.0	13.0	12.0	11.0
90th percentile	13.0	12.0	13.0	14.0	14.0	17.0	13.0	13.9
95th percentile	16.0	14.1	16.0	15.9	15.0	19.2	18.0	15

*M = Mean, *SD* = Standard Deviation, *Mdn* = Median.

Comparing mean and median for each subscale of the SDQ for boys and girls at different ages indicates a slight skewness in data. Skewness is varying between −1.2 and 1.5 in most cases, and also up to 1.9 in just a few instances. The kurtosis varied between −0.4 and 1.6, but in the case of the subscale Conduct Problems it did reach high levels, up to 7.8 in case of 11-years old girls.

## Discussion

Use of questionnaires such as the SDQ is a cost-effective way of collecting data from different informants. Data from such instruments might be a good starting point for a decision to collect more data for selection and intervention purposes. However, the usefulness of these instruments is dependent on their psychometric properties and availability of norms from the general population.

The psychometrics of the SDQ in previous research have been fair to good given the mixed finding in reproducing its original factor structure. In total, satisfactory internal consistency and test-retest reliability have been reported (for a review, see [20-22,39,40]). The present study is the first one to provide more information on the psychometrics of the SDQ when administered online. Given the large skewness and kurtosis of three of its items, polychoric ordinal alpha was used to investigate the internal consistency. The SDQ subscales showed good to excellent reliability, ranging from .85 to .91. This is higher than what other studies have reported e.g., .57 to .77 [32], .58 to .76 [41] and .59 to .80 [42]. These studies have not used polychoric ordinal alpha, but instead Cronbach’s alpha, which might explain some of the differences. The reported internal reliability pattern is very similar across the studies, Hyperactivity-Inattention subscale has the strongest reliability and Peer Problems subscale has the weakest. The SDQ correlated significantly in expected direction with the DBD, which supports the validity of the SDQ. In addition, the confirmatory factor analysis of data obtained from online administration of the SDQ resulted in an excellent fit. The validity of the five-factor model was supported, which supports its construct validity.

Norms for the SDQ (parent version) for the targeted age of children is scarce. In a previous validation study of the SDQ [43] data were obtained from 263 randomly selected parents of children 5–15 years old in the general population, but the authors did not present the norms in detail besides a figure presenting mean value of the Total Difficulties score of SDQ and subscales for the entire sample. Comparing norms from the present study to an Australian study [42] with somewhat younger children shows good similarities, with the exception of slightly lower mean value of reported Emotional Symptoms in the present study for girls (1.6) compare to the Australian study (2.0). The same pattern emerged in comparing norms for boys in the current study to the Australian norms. Present norms correspond very well with those obtained from other Scandinavian countries as well (for a review, see [44]). Comparing the central tendency figures in the present study with those obtained in Denmark (Aarhus) and Norway (Akershus), shows that data in the present study fit slightly better with the Norwegian norms, and is slightly higher than the Danish norms. However, the differences are small and the pattern of data in the present study confirms the conclusion made by Obel et al. (2004) that the SDQ scores are very similar across Nordic countries. An important finding in the present study is that norms obtained from online administration of the SDQ are very similar to those using paper-and-pencil. This study therefore supports the idea that the SDQ can be used for online data collection without any concerns about its psychometrics or how the obtained scores can be interpreted in relation to the norms from the general population.

The present study had some limitations that are worth mentioning for future replications. The first was a larger response through paper-and-pencil condition than via the Internet, instead of a fairly equal response rate. However, we did not find any differences between those responding via the Internet compared to those who did it by using paper-and-pencil. The parent’s educational level is slightly higher than the average educational level of parents in Sweden. This seems to be a common bias in research studies. In other words, parents with higher education are more willing to participate in such studies than those with lower education. Nevertheless, we found no substantial differences (i.e., with at least medium or close to medium effect sizes) on the SDQ between respondents with higher versus lower education. The percentage of girls and boys at each age interval was also compared to the national data retrieved from the Statistics Sweden (http://www.scb.se) on all girls and boys aged 10–13 years. At each age interval in the total population, about 48.5% are girls and 51.5% boys. In the study sample the mean percentage of girls at these ages is about 51% (boys 49%). Gender distribution of boys and girls in the sample might thus be viewed as representative of the general population of children at these ages. The response rate (just above 51%) was low despite several reminders and some incentives to increase the response rate. It would also have been informative to have some data on the age of the responding parents. Unfortunately this parameter was not included in the questionnaire. Lack of information regarding the characteristics of children (e.g., native language, order and number of siblings) is another limitation of the study. Such information would have been helpful for investigating the representativeness of the sample in relation to national data. Finally, some studies have shown that those who do not respond to surveys regarding psychological problems tend to suffer from such problems to a higher extent than those who are more willing to participate (e.g., [45]). Translated into the context of the current study, parents whose children exhibit some conduct problems might be less willing to participants in studies such as the current one. On the other hand, obtained norms in our study are in line with norms based on studies with less drop-out. Nevertheless, our findings need to be interpreted with caution as a risk for positive selection cannot be ruled out. As the participating parents reported a higher level of education than the general population, and a larger portion of them were married, available norms from the present study should also be viewed in light of these limitations.

## Conclusions

The present study is the first to provide data on the psychometrics of the SDQ when administered online and it makes the SDQ more useful in the context of screening, assessment and evaluation of interventions due to provision of norms from the general population.

## Consent

Written informed consent was obtained from all the participants.

## Competing interests

The authors declare that they have no competing interests.

## Authors’ contribution

AB contributed to data analysis and interpretation, and drafted the first version of the manuscript. PE was involved in conceptualizing and designing the study, data analysis and interpretation as well as drafting and editing the manuscript. AG contributed to the conception and design of the study, data collection, data analysis and interpretation, as well as drafting and revising the manuscript. All authors read and approved the final manuscript.

## Authors’ information

AB is a Ph.D. candidate, and clinical psychologist, and licensed psychotherapist. PE is Med. Dr., clinical psychologist and licensed psychotherapist. Her major research area is about psychopathology and treatment of disruptive behavior among children and adolescents. AG (Ph.D.) is a professor of clinical psychology, clinical psychologist and licensed psychotherapist. His main focus in research is on psychopathology, prevention and treatment of eating disorders and conduct problems.
